# Lidocaine Cyclodextrin complex Ophthalmic Drop, a New Topical Anesthetic Choice

**Published:** 2012-09-30

**Authors:** AA Sabermoghadam Ranjbar, O Rajabi, R Salari, H Ashraf

**Affiliations:** 1 Ophthalmology research center, Mashhad University of Medical Sciences, Mashhad, Iran; 2 Department of Medicinal Chemistry, School of Pharmacy, Mashhad University of Medical Sciences, Mashhad, Iran; 3 Orthopedics research center, Mashhad University of Medical Sciences, Mashhad, Iran

**Keywords:** Drug Delivery, Lidocaine, Cyclodextrin, Ophthalmicdrop, Symblepharon, Pterygium, Dysport

## Abstract

**Background:**

Topical anesthesia is a safe and cost-effective method considered as the first-choice in many procedures. Due to the physiological characteristics of eye, most of the local anesthetics cannot efficiently penetrate through the conjunctiva deep to tenon. The aim of this pilot study was to find a new form of lidocaine to give a sufficient level of anesthesia.

**Methods:**

Lidocaine Cyclodextrin complex ophthalmic drop was produced and its pharmacological properties were studied [tested] in standard temperature and pressure. 30 patients (18 males, 12 females) with the mean age of 30.68±8.02 years enrolled in this clinical trial. All the patients were fully informed and signed the ethics committee consent forms. The patients were given tetracaine drop as the anesthetic: 3 drops separated 2 minute apart 10 min before the intervention. If we achieved a sufficient level of anesthesia, the procedure was done after. If the patient could not tolerate the procedure, the method was changed to lidocaine drop (administered after wash-out period like the first drop).The last option was conventional injection method if the patient could not tolerate the procedure with the second method either.We used this type of anesthesia for conventional procedures such as forced duction test, symblepharon, pterygium, and disport injection into extra-ocular muscles. All the procedures were done by one surgeon in a university hospital. We used a 0 to 10 visual analogue scale for pain and two 0 to 4 patient and physician satisfaction scales designed for this study.

**Results:**

The mean pain score was 7.53±0.90 in group 1 and 3.03±1.83 in group 2 (P=0.00). Patient and surgeon satisfaction in group 1 were 1.33±0.48 and 1.40±0.56 respectively; while 3.23±1.00 and 3.56±0.77 for group 2 (P=0.00). Tetracaine drop could not induce sufficient anesthesia for none of the patients. Cyclodextrin based lidocaine drop was successful except For two patients for whom we changed the anesthesia to Sub-conjunctival injection method.

**Conclusion:**

Our newly manufactured cyclodextrin based lidocaine eye drop could successfully induce sufficient anesthesia for 28 of 30 patients. Further studies with larger sample sizes are now being designed to find more clinical evidence about this method.

## Introduction

Premature medicine. Nowadays, lifestyle improvement and reduction of patients’ pain and suffer is one of the most important physicians’ goals.

Many superficial interventions are done under topical anesthesia in today's ophthalmology. Topical anesthesia provides a reversible inhibition of sodium channels in cellular level (1). Having low risk and being easily applicable grows the tendency among surgeons to use such methods and patients to accept them (2) However, available topical anesthetics are not capable of producing sufficient level of anesthesia for more invasive procedures.

One of the best ways to increase the efficacy of topical anesthetic is to change its pharmacodynamics. In other words, the topical anesthetic molecule should be changed in a way that improves its penetration through membranes covering the eye surface. In order to fulfill this, Cyclodextrin (which was applied for improving the penetration of other ophthalmic drugs before) was used as ocular penetration enhancer.

The traditional form of lidocaine which is easily accessible in the market is its hydrochloride salt form. Lidocaine hydrochloride is highly soluble in water; however, low penetration through the cell membrane is its problem mainly because of the ionization which leads to presence of positive ions on the amine group. To solve this, we should make the molecules as electrically neutral as possible but the neutral form of lidocaine is not soluble in water.

Cyclodextrins are a family of cyclic oligosaccharides, derived from glucose which has an internal space and can form a complex with many drugs. Internal surface of this space is hydrophobic and can let the non-polar molecules bind to it.(3) However, its external surface is extremely hydrophilic (because of hydroxyl groups) which makes the complex watery soluble.(4) Among the members of this family, hydroxyl propel beta-cyclodextrin has a good water solubility and its toxicity is not considerable compared to other members of this family.

There are some other ophthalmology drugs that were manufactured and tested with cyclodextrin complex in the literature, such as ketokonazol,(5) dexametazone, (6,7) asetazolamid,(8) and dorzolamid.(9)

In summary, the aim of this study was to manufacture a new topical anesthetic drop with better efficacy that makes these interventions possible. A method which can prevent injection methods’ risks while making less concern and discomfort for the patients.

## Materials and Methods

In this study complex of lidocaine (base) with hydroxyl propel beta cyclodextrin was prepared in watery environment and physiologic PH. Characterization of complex was approved by differential scanning calorimetery and spectroscopy (Fourier transform infrared spectroscopy (FTIR) methods). This complex has more solubility than lidocaine base in watery environment. Stoichiometric constant Characteristic of the complex was 1:1. This result was derived from phase solubility study.

The constant of complex composition was calculated and its value was41.3 M^-1^.The Complex had appropriate water solubility and its sterile solutions were expected to be more efficient than tetracaine hydrochloride for ophthalmic anesthesia.

### Complex preparation instruction

1. With the Stoichiometric ratio of 1:1, lidocaine and hydroxyl propel beta cyclodextrin were added to watery environment.

2. The complex was dried and ready to use by liofilizator.

3. Using phosphate buffer system (with concentration of 0.05M, pH=9), dry complex reached the appropriate volume.(4)

30 patients (18 males and 12 females) with the mean age of 30.68±8.02 years (all between 20 and 60 years old) who were ready for limited topical ophthalmologic interventions (forced duction test, Dysport injection into extra-ocular muscles, symblepharon, and pterygium surgery), were selected by the easy and target focused sampling method and enrolled in this pilot clinical trial. All the patients were fully informed and signed the ethics committee consent forms. If the patients felt any discomfort or if any complications occurred during the procedure, the method was changed to traditional injection method and any necessary treatment was done for the unwanted complications. Lidocaine cyclodextrin complex ophthalmic drop was produced and tested in standard conditions. Tetracaine Hydrochloride drop and Lidocaine cyclodextrin ophthalmic drop were labeled as the “solution 1” and “solution 2” in two similar bottles and were given to O.R. personnel. Patients were named as 1001 to 1030.

The patients were given tetracaine drop as an anesthetic, 3 drops separated 2 minute apart, 10 minutes before the intervention and then the procedure was performed if sufficient level of anesthesia was achieved. The pain score was measured from the beginning of the procedure. If the patient could not tolerate the procedure, the method was changed to lidocaine drop (administered after at least 30 minutes wash-out period like the first drop). The last option was Sub-conjunctival injection method if the patient could not tolerate the procedure with the second method either. All the procedures were done by one surgeon in standard conditions and in a university hospital. Reviewing the literature, we designed 3 scales for pain, patient satisfaction and physician satisfaction which were in a questionnaire with basic demographic and personal information (name, address, etc.). The validity and Reliability of questionnaire was approved by university psychology department.

We used a 0 to 10 visual analogue scale for pain, in which 0 means no pain and 10 refers to the patient’s worst pain experience(labor, renal colic, bone fracture, etc) And two 0 to 4 scales were designed for patient and physician satisfaction. Patients and physicians marked their satisfaction in a five choice question as the: and not at all, poor, average, good, excellent. According their response they give 0 to 4 satisfactory score respectively.

 Patients were fully informed before the study about the possibility of incomplete anesthesia, change in anesthesia method and potential complications of each method. All the steps were done under supervision of the regional ethics committee of Mashhad University of medical sciences.

 Data were analyzed with SPSS version 13 at the end of study. Results were shown in descriptive manner (as different tables, graphs, etc) and compared with each other. The kolmogorov-smirnov (KS) test approved the Normality of sample sizes and after this approval; the differences were examined in paired t-test for quantitative variables, and in (Chi-square) K2-test for qualitative variables. If the normality was not approved by KS test, the correct nonparametric tests were used.

## Results

The mean intervention time in pterygium patients was 14.53min with the standard deviation of 2.17 min. The mean intervention time for disport injection, symblepharon and forced duction test groups was 4.2±0.84min, 12±2.83min and 1.63±0.52min respectively.

 The mean patients’ pain score was 7.53±0.90 in group1 and 3.03±1.83 in group2 (P=0.00). Patient and surgeon satisfaction in group1 were 1.33±0.48 and 1.40±0.56 respectively; while being 3.23±1.00 and 3.56±0.77, for group2 respectively (P=0.00).

Tetracaine drop did not induced sufficient anesthesia for any of the patients. Lidocaine successfully induced local anesthesia except For 2 patients for which we changed the anesthesia to traditional injection method. Fortunately, we had no unexpected complications in this study. The results of pain, patient satisfaction, and surgeon satisfaction were analyzed and shown in table 1 regarding the type of intervention.

**Table 1:** The results of pain, patient satisfaction, and surgeon satisfaction regarding the type of intervention.

**Intervention**	**Drug group**	**Pain**	**Patient satisfaction**	**surgeon satisfaction**
Pterygium	Tetracaine	7.80±1.01	1.47±0.64	1.47±0.64
	Lidocaine	3.73±2.09	2.73±1.10	3.20±0.94
	P value	0.00	0.01	0.00
forced duction test	Tetracaine	7.25±0.71	1.25±0.46	1.25±0.46
	Lidocaine	2.13±0.99	3.88±0.35	3.88±0.35
	P value	0.00	0.00	0.00
symblepharon	Tetracaine	7.50±0.71	1.50±0.71	2.00±0.00
	Lidocaine	3.00±2.83	3.00±1.41	4.00±0.00
	P value	-	-	-
dysport injection	Tetracaine	7.20±0.84	1.00±0.00	1.20±0.44
	Lidocaine	2.40±1.14	3.80±0.45	4.00±0.00
	P value	0.00	0.00	0.00

Based on the study’s parallel findings and with conjunctival pinching exam, (10) a sufficient level of anesthesia was achieved while instilling 3rd drop of lidocaine. Some minutes later, just before the intervention began, the maximum anesthetic effect was achieved.

20 minutes after intervention began (30 min after the last drop), there was still efficient anesthesia in conjunctival pinching exam.

## Discussion

Making minimum pain and discomfort for the patients has always been one of the most important concerns of the physicians. Achieving this aim was one of the main reasons of Establishment and improvement of sedation methods, topical, local and general anesthesia methods. Since the discovery of cocaine anesthetic effects in 1884, there have been hopes and struggles on improvement and distribution of anesthetic drugs and techniques, which anticipated more facility and safety for future medical interventions.(11, 12)

Topical anesthesia involves instillation of anesthetic ophthalmic drop or similar drug forms on the corneal surface. The process is easy, rapid and cost-effective. Prevention of all the injection complications is the main advantage of this method, (13) which includes a wide range of complications and in worst case scenario, perforation of cornea, sclera, and visual loss.(14)

Using topical anesthesia is reported in many ophthalmic interventions. Thus, this method has its own limitations. Limited duration of effect, limited distribution of anesthesia to the cornea and surface of the glob (13) and free movement of eyelids and globe are the most important ones. (15, 16) Anesthesia may be incomplete. Regardless of the differences in details, more pain and discomfort has been reported in many studies that compared injection methods with topical anesthesia methods.(8,15,17)

We found no unwanted or allergic complications with our newly manufactured cyclodextrin based lidocaine eye drop. But the possibility was considered in all the stages of the study. It was described to the patient before the study, and the surgeon was ready for it.

Lidocaine-cyclodextrin ophthalmic drop was designed for efficient and long lasting anesthesia in invasive interventions and was successfully tested in the first step. The interventions in this study were forced duction test, symblepharon, pterygium and dysport injection.

It seems that lidocaine cyclodextrin ophthalmic drop can be a candidate for use in other interventions like chalasion, cataract, glaucoma/terabculectomy, vitrectomy, strabismus surgeries. Further studies are now being designed for such procedures.

It is important to bear in mind that our findings do not rule out the benefits of current anesthetics. Because of low cost, high availability and long history of application, tetracaine hydrochloride drop is still the first choice in many diagnostic or treatment procedures like extraction of foreign body.

Limitations: It was a pilot study with a small sample size which could be a backgrand for upcoming stronger studies. They could have more data to report. So then we could compare our findings with other studies and drugs.

We propose more studies with larger sample size and other control groups like lidocaine ophthalmic gel. At this level other variables could be measured like drug concentration, drug penetration, optimum time for beginning of anesthesia and duration of anesthesia.
